# Compulsory drug detention center experiences among a community-based sample of injection drug users in Bangkok, Thailand

**DOI:** 10.1186/1472-698X-11-12

**Published:** 2011-10-20

**Authors:** Joanne Csete, Karyn Kaplan, Kanna Hayashi, Nadia Fairbairn, Paisan Suwannawong, Ruth Zhang, Evan Wood, Thomas Kerr

**Affiliations:** 1 Heilbrunn Department of Population and Family Health, Mailman School of Public Health, Columbia University, 50 Haven Avenue, New York, NY 10032 USA; 2 Thai AIDS Treatment Action Group, 18/89 Vipawadee Road, soi 40 Chatuchak, Bangkok 10900 Thailand; 3 Department of Medicine, University of British Columbia, 2775 Laurel Street, 10th Floor, Vancouver, British Columbia V5Z 1M9, Canada; 4 British Columbia Centre for Excellence in HIV/AIDS, 608 - 1081 Burrard Street, Vancouver, British Columbia V6Z 1Y6, Canada; 5 Provincial Health Services Authority, 700 - 1380 Burrard Street, Vancouver, British Columbia V6Z 2H3, Canada

**Keywords:** Compulsory treatment, Thailand, injection drug use

## Abstract

**Background:**

Despite Thailand's official reclassification of drug users as "patients" deserving care and not "criminals," the Thai government has continued to rely heavily on punitive responses to drug use such as "boot camp"-style compulsory "treatment" centers. There is very little research on experiences with compulsory treatment centers among people who use drugs. The work reported here is a first step toward filling that gap.

**Methods:**

We examined experiences of compulsory drug treatment among 252 Thai people who inject drugs (IDU) participating in the Mitsampan Community Research Project in Bangkok. Multivariate logistic regression was used to identify factors independently associated with a history of compulsory treatment experience.

**Results:**

In total, 80 (31.7%) participants reported a history of compulsory treatment. In multivariate analyses, compulsory drug detention experience was positively associated with current spending on drugs per day (adjusted odds ratio [AOR] = 1.86; 95%CI: 1.07 - 3.22) and reporting drug planting by police (AOR = 1.81; 95%CI: 1.04 - 3.15). Among those with compulsory treatment experience, 77 (96.3%) reported injecting in the past week, and no difference in intensity of drug use was observed between those with and without a history of compulsory detention.

**Conclusion:**

These findings raise concerns about the current approach to compulsory drug detention in Thailand. Exposure to compulsory drug detention was associated with police abuse and high rates of relapse into drug use, although additional research is needed to determine the precise impact of exposure to this form of detention on future drug use. More broadly, compulsory "treatment" based on a penal approach is not consistent with scientific evidence on addressing drug addiction and should be phased out in favor of evidence-based interventions.

## Background

The United Nations estimates that about one-third of new HIV transmissions outside of sub-Saharan Africa are linked to injection drug use [1]. In some regions, including much of eastern Europe and parts of east and southeast Asia, contaminated injecting equipment is the source of the majority of new infections [2]. Ensuring access to sterile injecting equipment and to humane and scientifically sound treatment for drug dependence, including methadone maintenance therapy, should be central elements of HIV prevention in countries where injection drug use is linked to HIV transmission. Unfortunately, in many countries needle and syringe programs (NSP), including needle exchange, are politically unpopular and inaccessible for the majority of those who need it [3,4]. Relatively few countries make it a priority to ensure affordable and evidence-based treatment of drug dependence to all who need it [5].

Where illicit drug use is heavily criminalized, health services for people who inject drugs may be influenced or controlled by criminal law authorities. Treatment for drug dependence may be compulsory under the law. Various forms of compulsory or mandated drug dependence treatment, including drug courts, have been implemented in various settings. While evaluations have suggested some benefits of this type of approach, including reductions in drug-use-related criminal activity [6], a number of commentators have raised methodological concerns about existing evaluations (e.g., lack of data on post-release drug use, failure to incorporate intent-to-treat analyses, use of inappropriate control comparison groups) [7-11]. Further, recent reviews have suggested that the literature pertaining to compulsory or mandated treatment is highly inconsistent, and that this type of approach is less effective than voluntary treatment [8,12]. Concerns have also been raised about ethical issues related to compulsory treatment and the potential for associated human rights violations [10]. Still, there is some evidence indicating the benefits of offering integrating drug dependence treatment within criminal justice systems [6,13].

In a number of countries, including in southeast Asia, compulsory drug treatment includes prison-like detention and such practices as forced labor [14]. A recent WHO report examined compulsory drug detention centers in Cambodia, China, Malaysia, and Vietnam. Most centers are operated by staff from the military or public security sector, although some centers (primarily those in Malaysia and China) include a small number of healthcare professionals (nurses, counselors, physicians). Most centers fail to employ evidence-based approaches for treating drug dependence and instead rely on forced detoxification (medically-assisted in some cases), labor, educational approaches, and physical exercise [14]. In a 2009 report to the UN Human Rights Council, the former UN Special Rapporteur on Torture underscored that non-consensual treatment for drug dependence violates both scientific and human rights norms [15]. Empirical data on the health impact of compulsory drug detention centers in the region are scant. Accounts from a number of Asian countries suggest that persons undergoing this punitive "treatment" suffer physical and psychological harms, as well as high rates of relapse to drug use [16,17].

Thailand has been widely praised for its response to HIV, which has resulted in demonstrable control of the epidemic in some population groups, including sex workers [18] . Sexual transmission of HIV declined by more than 80% in Thailand between 1991 and 2001 [19]. Among people who use illicit drugs, however, HIV prevalence has remained at about 40-50% over a long period [20,21]. Thailand has been criticized for failing to ensure access to NSP, methadone therapy, and other humane treatment for drug dependence [22]. In spite of a 2002 law that reclassified people who use illicit drugs as "patients" to be cared for, rather than criminals to be punished [23], the Thai government continues to rely heavily on compulsory drug detention--*bangkap bambat *or "forced treatment" in Thai--that almost always includes significant periods of prison-like detention [24,25]. Although the compulsory drug detention system was conceived as an alternative to incarceration, people mandated for this treatment are frequently detained in prison for about 45 days while their cases are being assessed [24,25]. A recent review revealed that the majority of the 84 centers in operation in 2008 were run by the Royal Thai Army, Air Force, or Navy [24]. Centers run by the military typically house between 100 and 400 individuals, while a smaller number of centers run by the Ministry of the Interior tend to house between 30 and 50 individuals. Individuals usually stay between three and six months in compulsory drug detention centers, although this period can be extended upon review. Activities within the centers typically involve intensive physical exercise akin to that found in military "boot camps," group work common among therapeutic communities, and vocational training. There have also been reports of cruel, inhuman, and degrading punishment within such centers [24]. From October 2008 to June 2009, there were an estimated 39,287 people in compulsory drug detention centers in Thailand [26].

There is little published information on the experience of compulsory drug detention in Thailand from the point of view of people living with drug dependence. Therefore, we sought to identify the prevalence and correlates of compulsory drug treatment exposure among a community-recruited sample of Thai people who inject drugs (IDU).

## Methods

### Participant Recruitment

The Mitsampan Community Research Project (MSCRP) is a collaborative research project involving the British Columbia Centre for Excellence in HIV/AIDS (Vancouver, Canada), the Mitsampan Harm Reduction Center (Bangkok, Thailand), the Thai AIDS Treatment Action Group (Bangkok, Thailand), and Chulalongkorn University (Bangkok, Thailand). In July-August 2008, the research partners undertook a cross-sectional study involving 252 community-recruited IDU. Participants were recruited through peer-based outreach efforts and word of mouth and were invited to attend the Mitsampan Harm Reduction Center to be part of the study. To be eligible to participate in this study, individuals had to have injected at least once in the previous six months. All participants provided informed consent and completed an interviewer-administered questionnaire eliciting demographic data as well as information about drug use, HIV risk behavior, interactions with police and the criminal justice system, and experiences with health care, including compulsory "treatment." Participants received a stipend of 250 Thai Baht (approximately US$7) upon completion of the questionnaire. The study was approved by the Research Ethics Boards of the University of British Columbia and Chulalongkorn University.

### Statistical Analyses

The primary outcome of interest in this analysis was a history of compulsory drug detention experience among IDU. We compared IDU who did and did not report a history of compulsory drug detention experience using univariate statistics and multivariate logistic regression. Variables considered included: median age (< 36.5 years vs. ≥ 36.5 years), gender, education level (up to secondary school vs. secondary school or higher), current employment (unemployed vs. employed), current illegal income generation (yes vs. no), average amount of money spent on drugs per day (> 300 vs. ≤ 300 Thai Baht or US$9), heroin injection ever (yes vs. no), methamphetamine injection ever (yes vs. no), methadone injection (i.e., illicit methadone use) ever (yes vs. no), overdosed ever (yes vs. no), use of drugs in combination (yes vs. no), syringe borrowing ever (yes vs. no), syringe lending ever (yes vs. no), methadone treatment use ever (yes vs. no), and reporting a history (yes vs. no) of drug planting by police (i.e., police have ever planted illicit drugs on one's person). To examine the bivariate associations between each independent variable and compulsory treatment experience, we used the Pearson χ^2 ^test. Fisher's exact test was used when one or more of the cells contained values less than or equal to five. We then applied an *a priori *defined statistical protocol by fitting a multivariate logistic regression model that included all variables that were significantly associated with compulsory drug detention experience at the *p *≤ 0.05 level in univariate analyses. All *p*-values were two-sided. We also investigated the prevalence of injection drug use in the past week among those who reported a history of compulsory drug detention. As well, we compared intensity of recent injection drug use (≥ daily injecting vs. < daily injecting) among those who did and did not report a history of compulsory drug detention experience using the Pearson χ^2 ^test.

## Results

In total, 252 IDU participated in this study; 66 (26.2%) were female, and the median age was 36.5 years. A total of 80 (31.7%) participants reported a history of compulsory detention. Table 1 presents the univariate analyses of factors associated with compulsory drug detention experience. Compulsory drug detention experience was positively associated with spending > 300 Thai Baht per day on drugs (odds ratio [OR] = 1.90; 95% confidence interval [CI]: 1.11 - 3.27), use of drugs in combination (OR = 1.99; 95%CI: 1.07 - 3.69), and ever having experienced drug planting by police (AOR = 1.99; 95%CI: 1.16 - 3.41).

**Table 1:** Factors associated with compulsory drug detention exposure among IDU in Bangkok, Thailand (*n *= 252)

Characteristic	Yes n = 80 (31.7%)	No n = 172 (68.3%)	Odds Ratio (95% CI)	*p - *value
**Median age**				
< 36.5 years	45 (56)	81 (47)	1.44 (0.85 - 2.46)	0.176
≥ 36.5 years	35 (44)	91 (53)		
**Gender**				
Female	23 (29)	43 (25)	1.21 (0.67 - 2.19)	0.529
Male	57 (71)	129 (75)		
**Education**				
≥ secondary	57 (71)	102 (59)	1.70 (0.96 - 3.01)	0.067
< secondary	23 (29)	70 (41)		
**Unemployed**				
Yes	16 (20)	30 (17)	1.18 (0.60 - 2.32)	0.625
No	64 (80)	142 (83)		
**Income from illegal sources**				
Yes	7 (9)	7 (4)	2.26 (0.77 - 6.68)	0.146
No	73 (91)	165 (96)		
**Median daily expenses for purchasing drugs**				
≥ 300 THB	49 (61)	78 (45)	1.90 (1.11 - 3.27)	0.019
< 300 THB	31 (39)	94 (55)		
**Ever injected heroin**				
Yes	78 (97)	156 (91)	4.00 (0.90 - 17.84)	0.051
No	2 (3)	16 (9)		
**Ever injected *yaba***				
Yes	54 (68)	107 (62)	1.26 (0.72 - 2.21)	0.416
No	26 (32)	65 (38)		
**Ever injected methadone**				
Yes	13 (16)	26 (15)	1.09 (0.53 - 2.25)	0.817
No	67 (84)	146 (85)		
**Ever used drugs in combination**				
Yes	63 (79)	112 (65)	1.99 (1.07 - 3.69)	0.029
No	17 (21)	60 (35)		
**Ever borrowed needles**				
Yes	32 (40)	57 (33)	1.35 (0.78 - 2.33)	0.289
No	48 (60)	115 (67)		
**Ever lent needles**				
Yes	29 (36)	63 (37)	0.98 (0.57 - 1.71)	0.954
No	51 (64)	109 (63)		
**Ever overdosed**				
Yes	30 (38)	45 (26)	1.69 (0.96 - 2.98)	0.067
No	50 (62)	127 (74)		
**Ever had drugs planted by police**				
Yes	48 (60)	74 (43)	1.99 (1.16 - 3.41)	0.012
No	32 (40)	98 (57)		
**Ever on methadone treatment**				
Yes	40 (50)	71 (41)	1.42 (0.83 - 2.42)	0.194
No	40 (50)	101 (59)		

Table 2 presents the multivariate analyses of factors independently associated with reporting a history of compulsory drug detention experience. As shown here, compulsory drug detention experience was positively associated with spending > 300 baht per day on drugs (adjusted odds ratio [AOR] = 1.86; 95%CI: 1.07 - 3.22) and reporting drug planting by police (AOR = 1.81; 95%CI: 1.04 - 3.15). In subanalyses, among those with compulsory drug detention experience, 77 (96.3%) individuals reported injecting in the past week. Intensity of recent injection drug use did not differ between those who were and were not exposed to compulsory drug detention (*p *> 0.14).

**Table 2:** Multivariate logistic regression analysis of factors associated with compulsory drug detention exposure among Thai IDU (*n *= 252)

Variable	Adjusted Odds Ratio (AOR)	95% Confidence Interval (CI)	*p - *value
**Ever used drugs in combination**			
(yes vs. no)	1.78	(0.94 - 3.36)	0.078
**Ever had drugs planted by police**			
(yes vs. no)	1.81	(1.04 - 3.15)	0.035
**Median daily expenses for purchasing drugs**			
(≥ 300 THB vs. < 300 THB)	1.86	(1.07 - 3.22)	0.028

## Discussion

Among a community-recruited sample of Thai IDU, almost one-third had experienced compulsory drug detention at some point. Having undergone compulsory drug detention was associated with having had drugs planted on one's person by the police, reporting greater spending on illicit drugs, as well as combination drug use (i.e., using more than one drug at a time). Virtually all (96.3%) of those who had undergone compulsory drug detention reported having injected drugs in the week prior to being interviewed for this study, and intensity of recent injecting behavior did not differ among those who were and were not exposed to compulsory drug detention.

Our finding of an association between compulsory drug detention experience and drug planting by police builds on a substantial body of literature demonstrating harms from and police corruption associated with drug enforcement policing [27,28], and raises concerns about the tactics used to force drug users into compulsory drug detention settings. This association may indicate that some individuals had drugs planted on them as police worked to meet quotas for arrest that were established as part of Thailand's state-sponsored "war on drugs" [29]. Alternatively, this association may reflect a breach of confidentiality in that police can identify and target individuals who have previously been in treatment. In any case, these findings indicate a need to investigate policing practices with an eye toward reform.

One possible explanation for our finding of high rates of active drug use among those IDU exposed to compulsory drug detention is that these individuals were more likely to be high-intensity drug users prior to being detained. Another potential explanation is that our sample was biased toward active drug users. To be eligible to participate in our study, individuals had to have injected only once in the past six months. Therefore, participants could have been exposed to compulsory drug detention and ceased injecting in the past six months and still been eligible to participate in the study. Still, our sample may be over-representative of those who relapsed after being in compulsory drug detention and may under-represent the population of IDU who ceased injecting following exposure to this type of program. However, is also possible that such centers may be failing to meet the stated goal of promoting reductions in or abstinence from drug use. It is notable that our results concerning post-compulsory drug detention drug use are strikingly similar to findings from evaluations of compulsory drug detention in China, which is estimated to have relapse rates of about 95% [16]. A World Health Organization (WHO) report suggests that the ineffectiveness of compulsory drug detention in Cambodia, China, Malaysia, and Vietnam is due not only to the lack of evidence-based practices in treating drug dependence but also to the lack of access to condoms and antiretroviral therapy in compulsory facilities [14]. Whatever its effectiveness, it is clear that compulsory drug detention in Thailand violates international norms. Noting that drug treatment "should not be forced on patients," WHO enjoins governments to limit compulsory treatment to "exceptional crisis situations of high risk to self or others" and specified periods of time [30]. In 2010, the executive director of the Global Fund to Fight AIDS, Tuberculosis and Malaria, Michel Kazatchkine, called for closure of compulsory detention of IDU under the guise of treatment and an end to the "repugnant abuses" in drug detention facilities [31]. The UN Special Rapporteur on Torture urged national governments to "ensure that their legal frameworks governing drug dependence treatment and rehabilitation services are in full compliance with international human rights norms" [15].

Exposure to compulsory drug detention was also associated with greater current expenditure for drugs. This could be explained by a selection effect, whereby compulsory drug detention selects for IDU who spend more on drugs, or alternatively, that being exposed to compulsory drug detention is associated with psychological sequelae resulting in greater spending on drugs and possibly higher intensity drug use (in this case, injecting or non-injecting).

As HIV among IDU remains a major public health problem in Thailand, the Thai government should be urged to concentrate on increasing access to proven means of HIV prevention in this population, including needle exchange and evidence-based treatment for drug dependence. Independent scrutiny of compulsory drug detention centers--that is, by investigators not linked to those running the treatment centers--is urgently needed, including of activities that may undermine the health or human rights of patients. It is an accepted principle, moreover, that no one treatment option works for all drug users [30]. The Thai authorities should focus on improving access to a range of humane and effective voluntary treatment options. Access to good quality methadone therapy, for example, remains very limited in Thailand [32,33].

This study is limited in several ways. The study sample of persons using the Mitsampan Harm Reduction Center was not randomly selected. It may therefore not be possible to generalize the findings of this study to Thai drug users more broadly. However, a strength of this study is its use of a community-recruited sample, rather than a sample selected from a treatment facility. The data are also based on self-report by drug users and may therefore be susceptible to response bias, including socially desirable responding. However, the participants in this study were blinded to the eventual use of this data; it is therefore unlikely that responses related to sensitive items (e.g., recent drug use) would be differentially reported by those with and without a history of compulsory drug detention. In addition, as noted above, the data do not permit detailed analysis of the compulsory drug detention experience that would enable recommendations for improving therapeutic elements or analysis of the timing of relapse post-treatment. Lastly, as mentioned above, we purposively selected a sample of IDU who had injected at least once in the previous six months, and therefore our findings pertaining to the potential effects of compulsory drug detention on recent drug use may be limited by selection effects. However, we note that our findings concerning rates of relapse are strikingly similar to findings observed in other settings [16].

## Conclusion

The stated policy of the Thai government that people living with drug dependence should be regarded as patients rather than criminals is in principle an important step toward an environment conducive to ensuring access to humane and effective health services for IDU. The findings of this study indicate that the principle is a long way from reality. The Thai government should phase out compulsory drug detention according to international recommendations, and in the immediate period should open all treatment facilities to independent scrutiny while working to remove barriers to voluntary, evidence-based health services for this neglected population.

## Competing interests

The authors declare that they have no competing interests.

## Authors' contributions

TK, KK, KH, PS, NF designed the overall study, RZ, TK, and EW designed and undertook the analyses specific to this manuscript, JC and KK prepared the first draft of the manuscript and all authors provided input on each draft of the manuscript. All authors read and approved the final manuscript.

## Pre-publication history

The pre-publication history for this paper can be accessed here:

http://www.biomedcentral.com/1472-698X/11/12/prepub
